# Impact of *PSCA* gene polymorphisms in modulating gastric cancer risk in the Chinese population

**DOI:** 10.1042/BSR20181025

**Published:** 2019-09-03

**Authors:** Kangpeng Yan, Kun Wu, Chao Lin, Zhigang Jie

**Affiliations:** 1 Medical College of Nanchang University, Nanchang 330000, Jiangxi, China; 2 Department of Surgery, Jiangxi Tumor Hospital, Nanchang 330000, Jiangxi, China; 3 Department of Surgery, First Affiliated Hospital of Nanchang University, Nanchang 330000, Jiangxi, China

**Keywords:** gastric cancer, polymorphism, PSCA, risk

## Abstract

Previous studies have identified the *prostate stem cell antigen* (*PSCA*) gene rs2294008 C > T and rs2976392 G > A polymorphisms to be associated with the risk of gastric cancer, the results of which are inconsistent. The present study aimed to evaluate the association between the two polymorphisms and the gastric cancer risk in the Chinese population. A hospital-based case–control study was conducted on 549 cases and 592 healthy controls. Odds ratios (ORs) and 95% confidence intervals (CIs) were applied to evaluate the association of the two polymorphisms on the gastric cancer risk. We found that both rs2294008 (CT vs. CC, OR = 1.55, 95% CI = 1.20–1.99, *P*<0.001 and CT+TT vs. CC, OR = 1.38, 95% CI = 1.09–1.74, *P*=0.008) and rs2976392 (GA vs. GG, OR = 1.61, 95% CI = 1.25–2.07, *P*<0.001 and GA+AA vs. GG, OR = 1.52, 95% CI = 1.20–1.92, *P*<0.001) were associated with an increased gastric cancer. In the combined analysis of the two polymorphisms, subjects with more than one risk genotype have a significantly increased risk of gastric cancer (OR = 1.38, 95% CI = 1.09–1.75, *P*=0.008) in comparison with those without any risk genotypes. In conclusion, our findings verified that the *PSCA* gene rs2294008 and rs2976392 polymorphisms were both significantly associated with an increased risk of gastric cancer in the Chinese population. Well-designed functional studies are to be warranted to confirm these findings.

## Introduction

Gastric cancer has become a major public health problem. In 2012, an estimated 951600 new gastric cancer cases and 723100 gastric cancer deaths occurred globally [1]. The highest gastric cancer incidence and mortality rates were found in East Asia, with approximately 679100 new cancer cases and 498000 cancer deaths reported in 2015 in China [2]. Carcinogenesis is a multi-step complex disease resulting from both environmental and genetic factors [3]. In Asia, the prevalence of *Helicobacter pylori* (*H. pylori*) is found as an important etiological factor for the development of gastric cancer [4]. In addition, previous studies showed that the potential environmental factors associated with gastric cancer might include tobacco smoking, alcohol use, dietary habits, unrefined vegetable oil, intake of water and daily consumption of meat-broth [5,6]. Furthermore, genetic factors have been also found to play an important role in the development of gastric cancer. In a two-stage genome-wide association study (GWAS) conducted in the Korean and Japanese populations, the *prostate stem cell antigen* (*PSCA*) gene polymorphisms were reported to be associated with an increased risk of gastric cancer [7].

The *PSCA* gene is located on chromosome 8q24.2, encoding a 123-amino acid glycoprotein, a cell surface antigen [8]. *PSCA* gene was identified as a prostate-specific antigen and to be associated with cell adhesion, proliferation and patient survival [9,10]. PSCA was reported to be overexpressed in many types of human cancers, and since it has restricted expression in normal tissues [11]. The *PSCA* gene polymorphisms were found to be associated with higher PSCA expression in cancer patients, such as rs2294008 [12]. Rs2294008 C > T and rs2976392 G > A polymorphism, the most widely studied polymorphisms in the *PSCA* gene, have demonstrated to be associated with an increased risk of gastric cancer [7]. However, rs2976392 was not found to be associated with the risk of cardia gastric cancer and rs2294008 was not associated with gastric cancer in the subsequent replication studies [13,14].

In view of this, we carried out a case–control study to investigate the association between *PSCA* gene rs2294008 C > T and rs2976392 G > A polymorphism and the risk of gastric cancer in the Chinese population.

## Materials and methods

### Study population

A total of 549 genetically unrelated cases with gastric cancer and 592 healthy controls of Chinese origin were included in the current study. All the patients were newly diagnosed and histopathologically confirmed primary gastric cancer, and recruited from the First Affiliated Hospital of Nanchang University from January 2010 to July 2014. In addition, age and gender-matched cancer-free Chinese controls were randomly recruited from the same region. Demographic characteristics and lifestyle habits of all subjects were collected. The current study was approved by the Institutional Review Board of First Affiliated Hospital of Nanchang University, Nanchang, Jiangxi, and carried out in accordance with the World Medical Association Declaration of Helsinki. All the participants have provided the written informed consent, accompanying with a donation of approximately 5 ml blood.

### Genotyping

Genomic DNA was extracted using the TIANamp Blood DNA Kit (TianGen Biotech Co. Ltd., Beijing, China) according to the manufacturer’s instructions. TaqMan real-time polymerase chain reaction (PCR) was applied to acquire the genotypes by using the ABI QuantStudio DX system [15,16]. Briefly, the final volume was 10 μl and consisted of 5 μl TaqMan universal PCR Master Mix, 0.5 μl Primer/probe mix and 10 ng genomic DNA. The PCR program had an initial denaturation step at 95°C for 10 min and 40 cycles at 94°C for 15 s and 60°C for 45 s. Allele frequencies were analyzed by using ABI SDS software. For quality control, genotyping was repeated on a random 10% of the samples, and 100% concordance rate was observed for the results.

### Statistical analysis

The Chi square test was applied to evaluate the differences in the distributions of genotypic frequencies and demographic variables among all the cases and controls. The odds ratios (ORs) and 95% confidence intervals (CIs), calculated by logistic regression models with adjustment for age, sex, smoking and drinking status, were used to assess the associations between the *PSCA* polymorphisms and the gastric cancer risk. The combined effect of the two *PSCA* polymorphisms and the gastric cancer risk was also calculated in logistic regression models. Moreover, we conducted stratification analyses by age, sex, smoking/drinking status and tumor site. All the statistics were performed by the SAS software (version 9.4; SAS Institute, Cary, NC). All the *P*-values were two sided, and *P*<0.05 was considered as statistically significant.

## Results

A total of 549 gastric cancer patients and 592 healthy controls were included in the present study. Subjects were well matched by age and sex. There were no significant differences between the cases and the controls for age (59.22 ± 10.94 vs. 58.45 ± 11.74, *P*=0.421) and sex (*P*=0.485). However, the controls were more likely to be smokers (*P*<0.001) and drinkers (*P*<0.001) compared with the cases of gastric cancer patients. Therefore, age, sex, smoking and drinking status were further adjusted for multivariate analysis.

The *PSCA* gene rs2294008 C > T and rs2976392 G > A genotype frequencies and their associations with the risk of gastric cancer in the Chinese population are shown in Table 1. For rs2294008 C > T, T is the risk allele. Additive model is a nonparametric regression method, is the trend of CC-CT-TT, dominant model is for CT+TT vs. CC and recessive model is for TT vs. CT+CC. For rs2976392 G > A, A is the risk allele. Additive model is the trend of GG-GA-AA, dominant model is for GA+AA vs. GG and recessive model is for AA vs. GA+GG. The *PSCA* gene rs2294008 C > T was found to significantly increase the risk of gastric cancer (CT vs. CC, OR = 1.55, 95% CI = 1.20–1.99, *P*<0.001 and CT+TT vs. CC, OR = 1.38, 95% CI = 1.09–1.74, *P*=0.008). A similar association with gastric cancer risk was also found for the *PSCA* gene rs2976392 G > A (GA vs. GG, OR = 1.61, 95% CI = 1.25–2.07, *P*<0.001 and GA+AA vs. GG, OR = 1.52, 95% CI = 1.20–1.92, *P*<0.001). When these two polymorphisms were combined, subjects with more than one risk genotypes have a significantly increased risk of gastric cancer (OR = 1.38, 95% CI = 1.09–1.75, *P*=0.008), compared with those without any risk genotypes.

**Table 1:** Association of *PSCA* genotypes with gastric cancer

Genotype	Cases (*n*=549)	Controls (*n*=592)	*P*^1^	Crude OR (95% CI)	*P*	Adjusted OR (95% CI)^2^	*P*^2^
rs2294008 (HWE < 0.001)							
CC	269 (49.00)	337 (56.93)		1.00		1.00	
CT	236 (42.99)	191 (32.26)		**1.55 (1.21–1.99)**	<0.001	**1.55 (1.20–1.99)**	<0.001
TT	44 (8.01)	64 (10.81)		0.86 (0.57–1.31)	0.482	0.86 (0.56–1.31)	0.482
Additive			<0.001	1.13 (0.94–1.34)	0.190	1.13 (0.94–1.35)	0.194
Dominant	280 (51.00)	255 (43.07)	0.007	**1.38 (1.09–1.74)**	0.007	**1.38 (1.09–1.74)**	0.008
Recessive	505 (91.99)	528 (89.19)	0.107	0.72 (0.48–1.08)	0.108	0.72 (0.48–1.08)	0.109
rs2976392 (HWE = 0.001)							
GG	267 (48.63)	349 (58.95)		1.00		1.00	
GA	236 (42.99)	192 (32.43)		**1.61 (1.25–2.06)**	<0.001	**1.61 (1.25–2.07)**	<0.001
AA	46 (8.38)	51 (8.61)		1.18 (0.77–1.81)	0.452	1.18 (0.76–1.82)	0.456
Additive			<0.001	**1.27 (1.06–1.53)**	0.009	**1.27 (1.06–1.53)**	0.009
Dominant	282 (51.37)	243 (41.05)	<0.001	**1.52 (1.20–1.92)**	<0.001	**1.52 (1.20–1.92)**	<0.001
Recessive	503 (91.62)	541 (91.39)	0.886	0.97 (0.64–1.47)	0.887	0.97 (0.64–1.48)	0.884
Combined effect of risk genotypes							
0	267 (48.63)	335 (56.59)	0.007	1.00		1.00	
1–2	282 (51.37)	257 (43.41)		**1.38 (1.09–1.74)**	0.007	**1.38 (1.09–1.75)**	0.008

Abbreviation: HWE, Hardy–Weinberg equilibrium.

1 Chi square test for genotype distributions between cases and controls.

2 Adjusted for age, sex, smoking and drinking status.

The risk genotypes were rs2294008 TT and rs2976392 AA.

Stratification analysis was further performed to evaluate the association between the two polymorphisms and gastric cancer risk under the dominant genetic model (Table 2). We found that subjects carrying rs2294008 CT/TT genotypes were associated with an increased risk of gastric cancer in the youngers (OR = 1.40, 95% CI = 1.00–1.94, *P*=0.049), the males (OR = 1.39, 95% CI = 1.06–1.84, *P*=0.019), the never-smoker (OR = 1.43, 95% CI = 1.03–1.97, *P*=0.031), the ever-drinker (OR = 1.60, 95% CI = 1.01–2.55, *P*=0.048), and the subjects with non-cardia tumor site (OR = 1.43, 95% CI = 1.11–1.85, *P*=0.006). For the *PSCA* gene rs2976392 G > A, we also found significantly increased associations in the subgroups of ≤59 years old (OR = 1.53, 95% CI = 1.10–2.13, *P*=0.012), >59 years old (OR = 1.48, 95% CI = 1.05–2.08, *P*=0.024), males (OR = 1.56, 95% CI = 1.18–2.06, *P*=0.002), never and ever smoking (OR = 1.52, 95% CI = 1.10–2.10, *P*=0.012 and OR = 1.56, 95% CI = 1.10–2.23, *P*=0.013), never and ever drinking (OR = 1.43, 95% CI = 1.09–1.89, *P*=0.010 and OR = 1.81, 95% CI = 1.13–2.8823, *P*=0.013), and non-cardia tumor site (OR = 1.55, 95% CI = 1.20–2.01, *P*=0.001). Furthermore, the combined effects of these two polymorphisms on increased risk of gastric cancer were also indicated in the younger subjects, the males, the never-smoker, the ever-drinker and the subjects with non-cardia tumor site.

**Table 2:** Stratification analysis for the association between *PSCA* polymorphisms and gastric cancer risk

Variables	rs2294008 (cases/controls)		Adjusted OR (95% CI)^1^	*P*^1^	rs2976392 (cases/controls)		Adjusted OR (95% CI)^1^	*P*^1^	Combined effect of risk genotypes (cases/controls)		Adjusted OR (95% CI)^1^	*P*^1^
	CC	CT/TT			GG	GA/AA			0	1–2		
Age												
≤59	132/137	139/130	**1.40 (1.00–1.94)**	0.049	131/177	140/124	**1.53 (1.10–2.13)**	0.012	131/170	140/131	**1.39 (1.00–1.94)**	0.049
>59	137/166	141/125	1.33 (0.95–1.86)	0.103	136/172	142/119	**1.48 (1.05–2.08)**	0.024	136/165	142/126	1.34 (0.95–1.97)	0.094
Sex												
Females	70/81	81/71	1.37 (0.86**–**2.16)	0.184	70/83	81/69	1.45 (0.91**–**2.29)	0.116	70/81	81/71	1.37 (0.86**–**2.16)	0.184
Males	199/256	199/184	**1.39 (1.06–1.84)**	0.019	197/266	201/174	**1.56 (1.18–2.06)**	0.002	197/254	201/186	**1.40 (1.06–1.84)**	0.018
Smoking status												
Never	162/159	174/122	**1.43 (1.03–1.97)**	0.031	162/163	174/118	**1.52 (1.10–2.10)**	0.012	162/158	174/123	**1.41 (1.02–1.94)**	0.039
Ever	107/178	106/133	1.35 (0.95**–**1.92)	0.095	105/186	108/125	**1.56 (1.10–2.23)**	0.013	105/177	108/134	1.38 (0.97**–**1.97)	0.072
Drinking status												
Never	213/227	218/178	1.31 (0.99**–**1.72)	0.055	212/235	219/170	**1.43 (1.09–1.89)**	0.010	212/225	219/180	1.30 (0.99**–**1.71)	0.064
Ever	56/110	62/77	**1.60 (1.01–2.55)**	0.048	55/114	63/73	**1.81 (1.13–2.88)**	0.013	55/110	63/73	**1.65 (1.04–2.64)**	0.034
Tumor site												
Cardia	75/337	66/255	1.20 (0.83**–**1.76)	0.337	73/349	68/243	1.39 (0.95**–**2.03)	0.087	73/335	68/257	1.26 (0.86**–**1.84)	0.230
Non-cardia	194/337	214/255	**1.43 (1.11–1.85)**	0.006	194/349	214/243	**1.55 (1.20–2.01)**	0.001	194/335	214/257	**1.41 (1.09–1.82)**	0.008

1 Adjusted for age, sex, smoking and drinking status.

The risk genotypes were rs2294008 TT and rs2976392 AA.

## Discussion

In this hospital-based case–control study, we investigated the association of the two *PSCA* gene rs2294008 C > T and rs2976392 G > A polymorphisms with the risk of gastric cancer in 549 patients and 592 healthy controls of Chinese origin. We found that the *PSCA* gene rs2294008 CT/TT and rs2976392 GA/AA genotypes significantly increased the risk of gastric cancer in the Chinese population, especially in the younger subjects, the males, the never-smokers, the ever-drinkers and the subjects with non-cardia tumor site. These findings suggested that the two *PSCA* gene rs2294008 C > T and rs2976392 G > A polymorphisms were associated with the gastric cancer risk and may contribute to the gastric cancer susceptibility.

**Figure 1:**
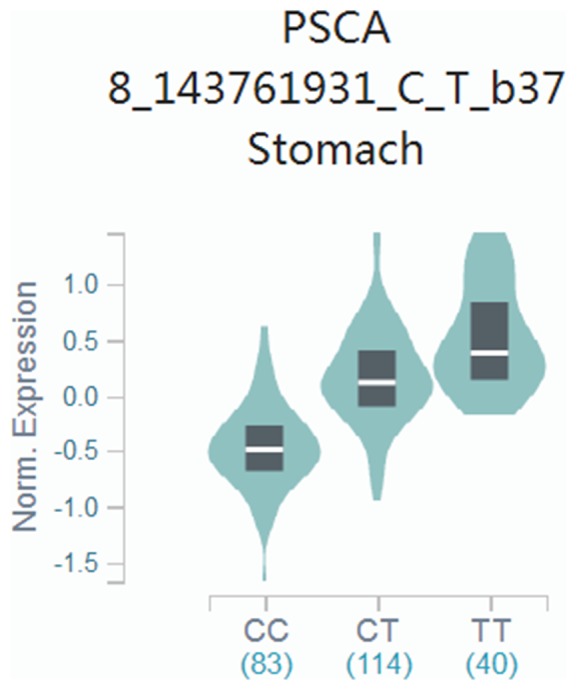
Effect of *PSCA* gene rs2294008 and mRNA expression based on the GTEx portal website Their relationship was the same as that between rs2976392 and mRNA expression.

**Figure 2:**
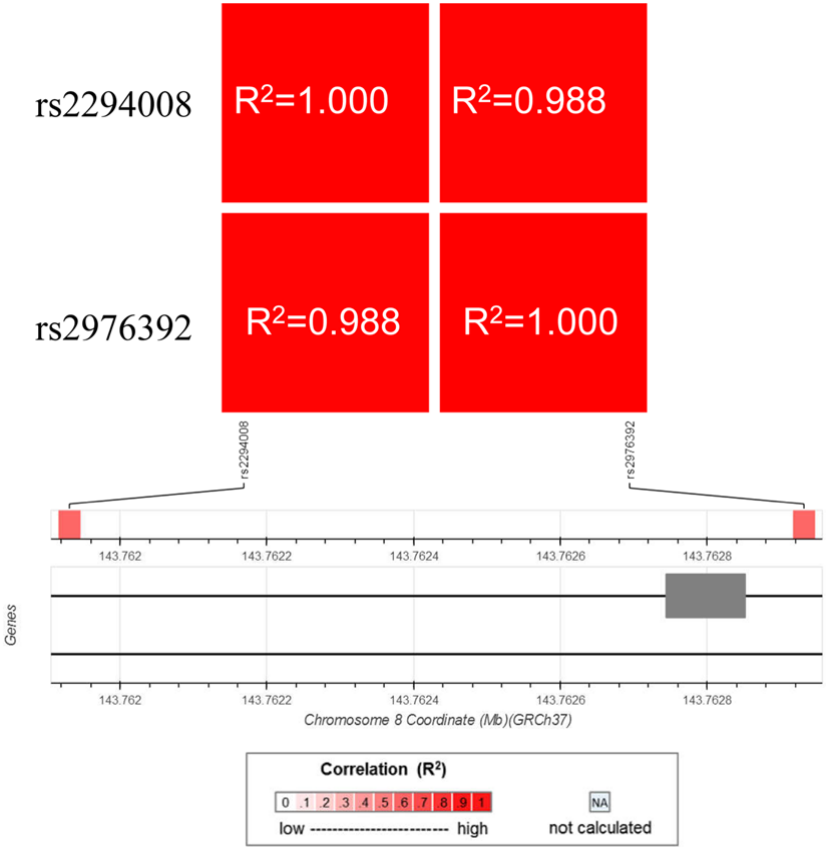
Linkage disequilibrium analysis for *PSCA* gene rs2294008 and rs2976392 in Han Chinese population consisting of CHB and CHS subjects

*PSCA* gene is a member of LY-6/Thy-1 family of cell surface antigens, and PSCA protein was reported as a cell surface marker having an important role in cell adhesion, proliferation and patient survival [8–10]. Abnormal expression of PSCA has been observed in many types of human cancers, including prostate, pancreas, bladder, ovarian, esophagus, gallbladder and gastric cancer [7,8,17–19]. Therefore, it is necessary to investigate the role of PSCA polymorphisms in the etiology of gastric cancer. Previously, GWASs had indicated that the two *PSCA* gene rs2294008 C > T and rs2976392 G > A polymorphisms were significantly associated with the risk of gastric cancer in the Chinese, Japanese, Korean and Caucasian populations [7,14,20]. However, the results remain controversial. For example, Ou et al. [21] found *PSCA* gene rs2294008 C > T polymorphism significantly associated with gastric cancer risk in Chinese populations, but such finding was not replicated in Lu et al. [14]. To further confirm the association, we conducted a replication study and found that the *PSCA* rs2294008 contributed to the increased gastric cancer susceptibility (CT vs. CC, OR = 1.55, 95% CI = 1.20–1.99, *P*<0.001 and CT+TT vs. CC, OR = 1.38, 95% CI = 1.09–1.74, *P*=0.008). The mechanism of *PSCA* rs2294008 associated with cancer risk remains unclear. However, *in vitro* experiments have noted that the variant might reduce the transcriptional activity of an upstream fragment of this gene [22]. Meanwhile, we confirmed the potential association between rs2294008 and mRNA expression doing the expression quantitative trait loci (eQTL) analysis with GTEx portal website (http://www.gtexportal.org/home/) (Figure 1). Whereas, for *PSCA* rs2976392, we also established that *PSCA* rs2976392 polymorphism is significantly associated with an increased risk of gastric cancer in the Chinese population. The *PSCA* rs2976392 polymorphism was also reported to relate to the increased risk of gastric cancer in some previous studies [16,23], but in some other studies including meta-analyses no such association was suggested [24]. This discrepancy should be due to the GWASs which had the large number and dominated the result of the pooled analyses in the meta-analysis [11]. Nevertheless, the molecular mechanism of *PSCA* rs2976392 was still unclear; its strong linkage disequilibrium with *PSCA* rs2294008 can explain the positive association in the previous case–control studies. Therefore, we also conducted linkage disequilibrium analysis for *PSCA* gene rs2294008 and rs2976392 in Han Chinese population consisting of CHB (Han Chinese in Beijing, China) and CHS (Southern Han Chinese) subjects (https://ldlink.nci.nih.gov/?tab=ldmatrix). As shown in Figure 2, there was significant linkage disequilibrium between *PSCA* rs2294008 and rs2976392 (*R^2^* = 0.988). In addition, we also examined the potential relationship between rs2976392 and the expression of mRNA, and found that their relationship was the same as that between rs2294008 and the expression of mRNA (Figure 1). In our current study, the combined effects of these two polymorphisms on the increased risk of gastric cancer were also found. It is similar to the effects of each individual genotype, mainly because of high linkage disequilibrium between *PSCA* rs2294008 and rs2976392.

Several limitations should be acknowledged in the present study. First, because of the inherent selection bias and information bias, we further conducted multivariate logistic regression analysis on age, sex, smoking and drinking status. Second, we only did the stratification analyses of age, sex, smoking, drinking status and tumor site, however, other gastric cancer risk factors, such as *H. pylori* infection, diet and occupation exposure, might also contribute to the etiological roles in gastric carcinogenesis. Third, we only investigated two polymorphisms in the *PSCA* gene. More potential polymorphisms are needed to be included. Finally, our findings gained from only the Chinese population and cannot be extrapolated to other populations.

In summary, our findings verified that the two *PSCA* gene rs2294008 C > T and rs2976392 G > A polymorphisms were significantly associated with an increased risk of gastric cancer in the Chinese population. Well-designed functional studies are to be warranted to confirm these findings.

## Abbreviations

CI: confidence interval
GTEx: genotype-tissue expression
GWAS: genome-wide association study
*H. pylori*: *Helicobacter pylori*
LY-6: lymphocyte antigen 6
OR: odds ratio
PCR: polymerase chain reaction
PSCA: prostate stem cell antigen
Thy-1: thymidylate synthase 1
